# Mammalian innate antiviral defenses: beyond interferon

**DOI:** 10.1128/jvi.01707-24

**Published:** 2025-10-20

**Authors:** Emily A. Rex, Joy M. Shaffer, Daniel M. Deng, Don B. Gammon

**Affiliations:** 1 Department of Microbiology, University of Texas Southwestern Medical Center 12334, Dallas, Texas, USA; New York University Department of Microbiology, New York, New York, USA

**Keywords:** interferon-independent antiviral immunity, innate immunity, TRIM E3 ubiquitin ligases, FEAR pathway, autophagy, septins, programmed cell death, viral immune evasion

## Abstract

Mammalian cells employ a wide array of antiviral defense mechanisms to restrict viral replication at virtually all steps of the viral life cycle. Notably, the interferon (IFN) response has been shown to play a central role in restricting the replication of disparate viral pathogens in mammals. Consequently, since its discovery in 1957, the IFN response has dominated antiviral immunity research, leaving IFN-independent pathways relatively understudied. Exploring these alternative host defenses is crucial for understanding the complete arsenal that mammalian hosts deploy to combat viral disease, as IFN responses undoubtedly work in concert with other antiviral defenses to achieve virus restriction. Here, we discuss selected examples of antiviral factors and pathways in mammals that are not classically associated with the IFN response. These defenses range from constitutively expressed host restriction factors that directly inhibit specific steps of the viral life cycle to signaling pathways that invoke IFN-independent antiviral gene expression programs to cell death mechanisms that sacrifice the infected cell to prevent viral spread. Ultimately, our goal is to highlight the diversity of IFN-independent antiviral defenses that mammalian hosts utilize to block viral infection.

## INTRODUCTION

Interferons (IFNs) are classified into three groups based on the different receptors they bind and have been reviewed in detail elsewhere (1–4). Here, we will focus on discussing the type I IFN (IFN-I) response as it has long been regarded as the most vital antiviral defense system in mammals, where it induces a plethora of IFN-stimulated genes (ISGs) that encode factors which restrict virus replication through distinct mechanisms (1, 5, 6). The importance of the IFN-I response is underscored by the strong association of animals and humans with deficiencies in IFN-I responses with increased susceptibility to viral infection (6–9). Moreover, virtually all mammalian viruses encode antagonists of the IFN-I response, further highlighting the central role of the IFN-I response to the evolutionary battle between viral pathogens and their mammalian hosts (10).

Accumulating evidence suggests that mammals also employ a diverse set of IFN-I-independent antiviral defenses that likely operate alongside IFN-I responses. Many of these alternative defenses are ancient innate responses that predate the rise of IFNs in jawed vertebrates during eukaryotic evolution but have been retained in mammalian hosts to combat infection (11). We define IFN-I-independent antiviral immunity as defenses that do not rely on canonical IFN or ISG production or those that induce ISGs but through non-canonical signaling pathways (1, 2). Such responses are likely critical to the overall control of viral infections, particularly when viruses deploy effective IFN-I antagonism mechanisms (2, 12). Moreover, IFN-I-independent responses can function in the absence of parallel IFN-I responses, which can be especially critical in stem cells where IFN-I signaling is incongruent with the maintenance of stem cell pluripotency (13). IFN-I-independent defenses include both constitutive and inducible cell-intrinsic restriction mechanisms that attempt to block viral replication at various stages of the viral life cycle or that activate programmed cell death to sacrifice infected cells to prevent further viral spread. *In vivo*, IFN-I-independent responses can also promote the recruitment of immune cells to the sites of infection and thus can function to activate other arms of the immune system (14). Understanding IFN-I-independent antiviral responses is not only critical for ascertaining a more complete view of mammalian innate immunity but also may offer novel therapeutic strategies for the treatment of viral diseases, which is important given the adverse side effects of IFN therapy (15, 16).

Here, we discuss selected examples of IFN-I-independent antiviral defenses in mammals, focusing on the molecular mechanisms by which they restrict viral replication, the cellular and viral contexts in which these defenses operate, and their contribution to the outcome of viral infection *in vivo*. While not the focus of this article, we also provide examples of viral evasion strategies to overcome the discussed IFN-I-independent responses to illustrate their physiological relevance to the evolutionary struggle between viral pathogens and their mammalian hosts. Importantly, due to space limitations, many additional IFN-I-independent antiviral defenses that also combat viral disease (see Table 1 for additional examples) are unable to be discussed here in detail. Thus, the examples discussed below are meant to illustrate the diversity of IFN-I-independent defenses employed by mammals rather than provide a comprehensive overview of all known IFN-I-independent antiviral mechanisms.

**TABLE 1:** Additional examples of antiviral defenses that can function independently of IFN-I signaling^*a*^

Host antiviral factor/response	Putative mechanism	Reference
APOBECs	Cytidine deamination (editing) of viral nucleic acids; editing-independent mechanisms	(17, 18)
SAMHD1	Reduction of intracellular dNTP pools; inhibition of viral capsid nuclear import; inhibition of lipid biosynthesis	(19–22)
SAMD9	Cleavage of phenylalanine-encoding tRNA and translation inhibition	(23–26)
Integrated stress response	Translation inhibition; induction of autophagy or programmed cell death	(27)
RNA interference	Cleavage of viral RNA	(28–30)
MARCH proteins	E3 ubiquitin ligases that promote degradation of viral glycoproteins	(31–33)
Reactive oxygen species	Interference with virion morphogenesis	(34)
PJA1 and Structural Maintenance of Chromosomes complex	Silencing of viral episomal DNA	(35, 36)

^*a*^ Expression of some of the factors listed can be further upregulated by IFN-I signaling.

## EXAMPLES OF IFN-I-INDEPENDENT ANTIVIRAL DEFENSES IN MAMMALS

### ISG-mediated antiviral defense independent of canonical IFN-I signaling

ISGs are classically thought of as being induced by canonical IFN-I signaling involving IFN-α/β binding to the IFN-α/β receptor (IFNAR), activation of intracellular kinases (JAK1, JAK2, and TYK2) that phosphorylate STAT1/STAT2 transcription factors, and the translocation of STAT1/STAT2/IRF9 (ISGF3) complexes into the nucleus to induce transcription of ISGs containing IFN-stimulated response elements (ISREs) (1, 37). However, there is substantial evidence that basal levels of ISGs or induction of ISGs through mechanisms independent of JAK-STAT signaling can provide initial viral containment, and such early ISG responses may be less susceptible to viral evasion strategies (see more comprehensive reviews on this topic reviewed in references 2, 38).

One mechanism for IFN-I-independent ISG induction involves recognition of pathogen-associated molecular patterns (PAMPs) by host-encoded pattern recognition receptors (PRRs) such as RIG-I, MDA5, and cGAS, which leads to the activation of IFN regulatory factors IRF3 and IRF1 (39, 40). These transcription factors can then bind to ISREs in the promoters of ISGs and initiate their expression without requiring IFN secretion or STAT activation (2). For example, cGAS can activate a STING-IRF3 signaling axis to induce ISGs in STAT1^-/-^ fibroblasts (39, 41), and human cytomegalovirus (HCMV; *Herpesviridae*) infection can induce robust, IRF-3-dependent ISG expression when JAK-STAT signaling is abrogated by viral STAT1 antagonists (2, 41). Interestingly, MAVS, which is known to associate with mitochondria to mediate canonical, IRF-3-dependent IFN-I responses downstream of RIG-I and MDA5, can also associate with peroxisomes to activate IRF-1-dependent ISG induction that does not require JAK-STAT signaling (42). Additionally, experiments using viral RNA mimetics or UV-inactivated viruses demonstrate that cells can mount rapid ISG responses via IRF1 or IRF3, independently of IFN cytokine production or JAK-STAT signaling (43–45). Moreover, MDA5, a sensor of viral dsRNA, was implicated in restricting coxsackievirus B3 (CVB3; *Picornaviridae*) replication in murine fibroblasts independently of IFNAR signaling (46). These examples illustrate how upstream components of IFN-I signaling pathways (e.g., PRRs and IRF3/IRF1) can play critical roles in mounting ISG induction without the requirement for JAK-STAT signaling.

Additionally, non-canonical cytokine pathways can also induce ISGs independently of IFN-α/β binding to IFNAR. The cytokines TNF-α, IL-1, and IL-27 can drive ISG expression through the action of various transcription factors, including NF-κB, STAT1, and IRF-3 in the absence of canonical IFN-I signaling (47–50). The physiological relevance of NF-κB-mediated antiviral responses to controlling viral infection is well illustrated by the finding that vaccinia virus (VACV; *Poxviridae*) encodes at least 18 independent inhibitors of NF-κB signaling (51). Importantly, ISGs induced through these IFN-independent mechanisms can play critical, early roles in restricting viral replication. For example, in severe acute respiratory syndrome coronavirus 2 (SARS-CoV-2) (*Coronaviridae*)-infected hamsters, lung epithelial cells significantly upregulate ISGs (e.g., MX1, IFIT2, and IFIT3) prior to detectable IFN production, and this corresponds with early containment of viral replication (52).

In summary, IFN-I-independent induction of ISGs via IRF3/IRF1 activation and alternative cytokine pathways can be crucial in mounting early innate antiviral responses. These mechanisms enable cells to rapidly deploy antiviral defenses that can be sufficient to control viral infection under low multiplicity of infection conditions (53). Moreover, these alternative ISG induction mechanisms both bide time for the host to robustly induce ISGs through canonical IFN-I signaling and provide new routes for ISG induction that viral factors may not fully antagonize.

### Direct ubiquitination of viral proteins by tripartite motif-containing 7

Tripartite motif-containing 7 (TRIM7) (also known as GNIP or RNF90) is a member of the TRIM family of E3 ubiquitin ligases that has received considerable attention in recent years due to emerging evidence suggesting it plays diverse, context-dependent antiviral and proviral roles during viral infection. Here, we focus on discussing the direct antiviral role of TRIM7 in mediating viral protein degradation; however, TRIM7 has also been implicated in both negative and positive regulation of innate immune responses, including IFN-I and NF-κB pathways (reviewed in references 54, 55).

Early evidence for an antiviral role of TRIM7 emerged from CRISPR activation screening that identified TRIM7 as a restriction factor for murine norovirus (MNoV; *Caliciviridae*) in human and murine cells (56). TRIM7 proteins encode a conserved N-terminal “RBCC” motif (composed of RING, B-box, and coiled-coil domains) and a C-terminal PRYSPRY domain that confers substrate specificity (55). Importantly, TRIM7 is not induced by IFN-I signaling and thus is not an ISG (57). The TRIM7 gene encodes four isoforms, with isoform 1 (the canonical and longest isoform that encodes a C-terminal PRYSPRY domain) and isoform 4 (a shorter isoform lacking the PRYSPRY domain) being the most well-studied isoforms and the only isoforms that encode the RING domain required for E3 ubiquitin ligase activity (56). Interestingly, overexpression of TRIM7 isoform 1, but not isoform 4, restricted MNoV replication in murine and human cells (56), suggesting an important role for the PRYSPRY domain. Transfection of MNoV genomic RNA into TRIM7-overexpressing cells was unable to overcome the block to MNoV replication, suggesting that TRIM7 inhibited MNoV replication post-entry (56). Shortly after this report, an independent study screened a library of 118 RING-type E3 ubiquitin ligases for antiviral activity and identified TRIM7 as a potent cell-intrinsic restriction factor for a wide array of human enteroviruses, such as CVB3 (58). However, TRIM7 did not affect flaviviruses, alphaviruses, or paramyxoviruses (58). These observations indicated that TRIM7 has selective antiviral activity against members of *Caliciviridae* and *Picornaviridae*.

Subsequent structural and biochemical studies revealed that TRIM7 utilizes its PRYSPRY domain to specifically recognize proteins terminating in C-terminal glutamine residues as substrates (59–61). Importantly, C-terminal glutamine-terminated proteins are often generated during the replication of several positive-sense ssRNA viruses, such as caliciviruses and enteroviruses, that use viral 3C-like proteases to cleave their polyproteins to generate viral proteins ending in glutamine (54, 55, 59). Once bound, TRIM7 catalyzes K48-linked polyubiquitination via its RING domain, leading to the proteasomal degradation of targeted viral components (54, 55). For example, TRIM7 targets the CVB3 2BC protein, a membrane remodeling factor, for ubiquitination and degradation in the proteasome (58). Interestingly, TRIM7 could bind to both viral 2C and its precursor 2BC, but TRIM7 only mediated degradation of the latter, revealing an unappreciated vulnerability of viruses to host restriction via targeting of their precursor proteins (58). Two recent studies have demonstrated that MNoV NS6 proteins, which terminate at a glutamine after viral protease cleavage, were targeted by TRIM7 (59, 62). Interestingly, in contrast to the finding with enteroviruses, TRIM7 could only bind to NS6, but not the NS6-NS7 precursor protein (62).

Given the potent antiviral activity of TRIM7, several viruses have evolved countermeasures to evade TRIM7-mediated restriction. For example, the 3C proteases from CVB3 and poliovirus (*Picornaviridae*) cleave TRIM7 at a conserved glutamine site (Q24), which dampens its E3 ubiquitin ligase and antiviral activities (63). However, these viral evasion strategies are clearly insufficient to completely inactivate TRIM7 function because serial passage of CVB3 in TRIM7-overexpressing cells gave rise to variants encoding a T323A point mutation in 2C that resulted in further TRIM7 resistance (58). Notably, these CVB3 variants exhibited enhanced replication and virulence in mice (58), suggesting TRIM7 evasion has important consequences for enterovirus pathogenesis. Similar passaging studies using TRIM7-overexpressing cells with MNoV identified a TRIM7-resistant variant with a single F182C substitution that reduced cleavage of the NS6-NS7 precursor protein (62). This suggests that this mutant evades TRIM7 restriction by reducing the production of free NS6 that TRIM7 can target (62). However, in contrast to the CVB3 escape mutant, the F182C-encoding MNoV strain displayed highly attenuated replication and pathogenesis in mice (62). Other recent MNoV passaging studies selected for variants that overcome a post-entry restriction in human cells, and these mutants also displayed attenuated virulence in mice (64). These examples illustrate that there can be consequential evolutionary tradeoffs for overcoming host restrictions and highlight the importance of examining virus-host interactions in relevant *in vivo* models. This is further underscored by a recent study with TRIM7^-/-^ mice that has called into question whether TRIM7 plays a substantial role in antiviral defense against MNoV. Using two independently derived Trim7-deficient mouse strains, these authors found no differences in viral burden or tissue distribution of MNoV during either acute or persistent infections (65).

A recent study suggests that TRIM7 may also target SARS-CoV-2 proteins but through a non-degradative mechanism (66). TRIM7 ubiquitinated the SARS-CoV-2 membrane (M) protein at lysine-14 (K14), which suppressed M protein-mediated cell death induction that would typically promote viral spread (66). TRIM7^-/-^ mice exhibited greater weight loss and higher SARS-CoV-2 titers, suggesting an overall antiviral function for TRIM7 in mice (66). However, others have not found TRIM7 overexpression to affect SARS-CoV-2 replication in human HeLa cells (59); therefore, the physiological role of TRIM7 in SARS-CoV-2 infection remains unclear.

Interestingly, when TRIM7 engages with viral proteins, it does not always act as an antiviral factor. For example, during Zika virus (ZIKV; *Flaviviridae*) infection, TRIM7 was found to catalyze the K63-linked ubiquitination of the viral envelope protein, which enhanced virion infectivity and viral dissemination (67). Consequently, ZIKV replicated less efficiently in the brain and reproductive tissues of TRIM7^-/-^ mice (67). Thus, as with other TRIM family members (68), TRIM7 can play very different roles during viral infection, depending on the context.

### The FACT-ETS1-antiviral response pathway

**Fig 1:**
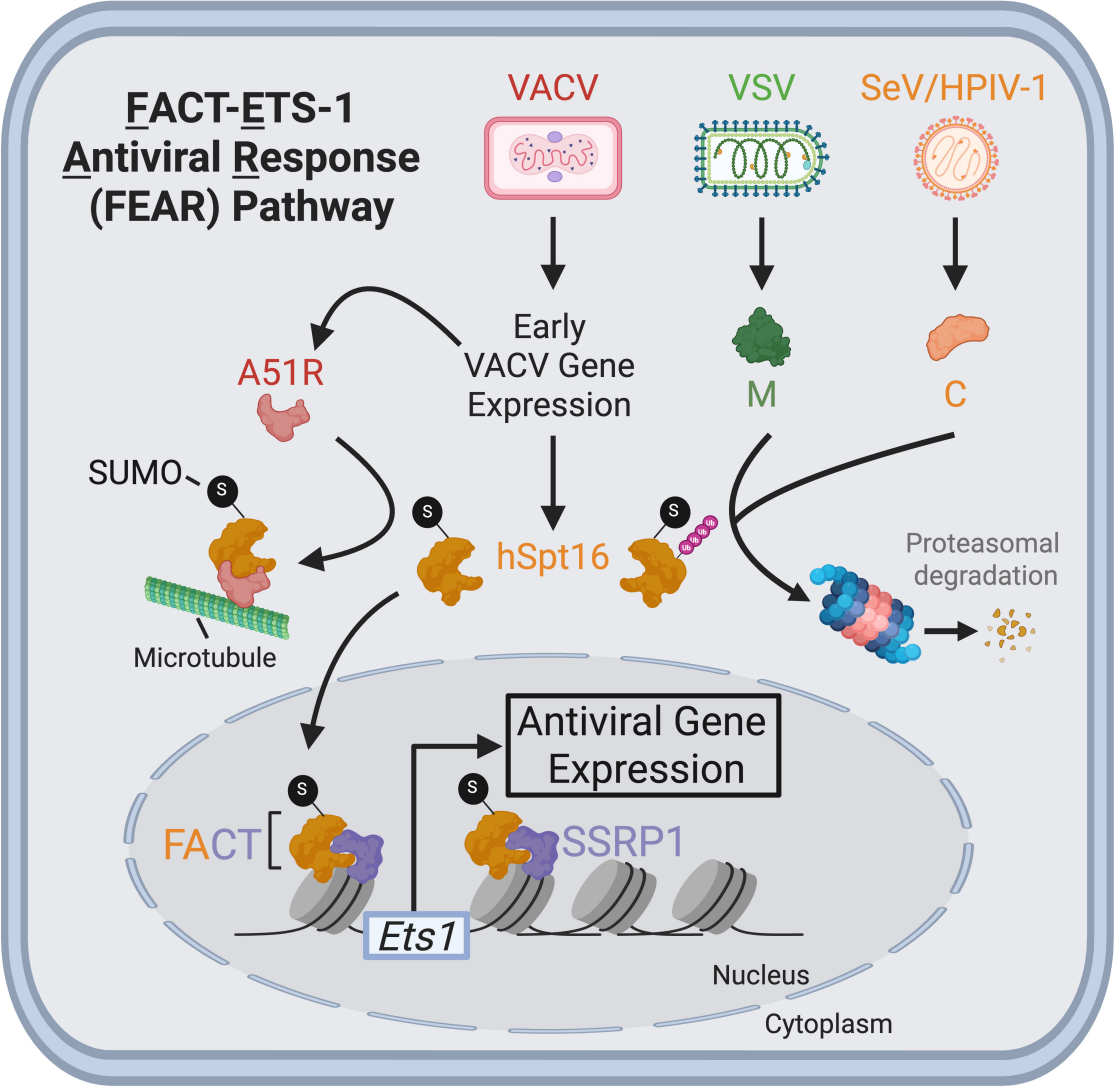
The FEAR pathway and its antagonism by DNA and RNA viruses. Upon VACV infection, early viral gene expression triggers SUMOylated hSpt16 proteins in the cytosol to translocate to the nucleus to form antiviral FACT complexes that induce the expression of the transcription factor ETS-1 for downstream antiviral gene expression. VACV-encoded A51R is an early protein that outcompetes SSRP1 for direct binding to SUMOylated hSpt16 and tethers cytosolic SUMOylated hSpt16 to microtubules, preventing its nuclear accumulation. In contrast, VSV-encoded matrix (M) and SeV- and HPIV-1-encoded C proteins promote the ubiquitination of SUMOylated Spt16, leading to its degradation in the proteasome. Figure adapted from references 73, 74 and created with BioRender.com.

The FACT-ETS1-antiviral response (FEAR) pathway is a newly discovered innate antiviral response that requires the “facilitates chromatin transcription” (FACT) complex, a histone chaperone that is highly conserved among invertebrate and vertebrate eukaryotes (69). The human FACT complex is a heterodimer composed of human suppressor of Ty 16 homolog (hSpt16) and structure-specific recognition protein-1 (SSRP1) subunits (70, 71). FACT regulates cellular gene transcription by remodeling histones in chromatin that would otherwise impede transcription complexes (72). The antiviral role of FACT was initially identified through studies with VACV. Upon VACV infection, early viral gene expression triggers a SUMOylated form of hSpt16 to translocate from the cytoplasm into the nucleus, where it forms specialized, antiviral FACT complexes with SSRP1. These antiviral FACT complexes subsequently bind chromatin to induce the expression of the transcription factor E26 transformation-specific sequence-1 (ETS-1), which is thought to invoke antiviral gene expression programs (73, 74) (Fig. 1). Though first identified to restrict VACV, a DNA virus, growing evidence suggests the FEAR pathway has a broader antiviral role against disparate RNA viruses, including vesicular stomatitis virus (VSV; *Rhabdoviridae*), influenza A virus (IAV; *Orthomyxoviridae*), yellow fever virus (*Flaviviridae*), and the *Paramyxoviridae* members Sendai virus (SeV) and human parainfluenza virus type 1 (HPIV-1) (73, 74).

ETS-1 is the founding member of the ETS transcription factor family that shares a common DNA-binding domain termed the “ETS domain” (75). Roles for ETS-1 in immune cell development have been reported previously (75–77), but ETS-1 had not been implicated in virus restriction. However, ELF1, another ETS transcription factor family member, was previously shown to induce an antiviral gene expression program that impedes DNA and RNA virus replication independently of the IFN-I response (78). Consistently, the FEAR pathway was activated in cells lacking key IFN-I signaling components, such as IRF3, IFNAR, or STAT1 (73, 74). Moreover, ISG induction was normal in ETS-1-deficient cells, suggesting that the FEAR pathway does not require or regulate the IFN-I response (74). Given that ETS transcription factors arose ~600 million years ago during invertebrate metazoan evolution, these transcription factors may activate ancient antiviral gene expression programs that predate IFN-I responses. This is consistent with the finding that the transcriptional programs induced by ELF-1 and the FEAR pathway were largely distinct from IFN-I transcriptional signatures (74, 78). However, key questions still remain regarding the identity of the host sensor(s) involved in activating the FEAR pathway and the ETS-1-induced factors that restrict viral replication (79).

The importance of the FEAR pathway to antiviral defense in mammals was further underscored by the identification of viral antagonists of this pathway. Poxvirus-encoded A51R proteins were the first FEAR pathway inhibitors described. A51R proteins outcompete SSRP1 to directly bind to cytoplasmic SUMOylated hSpt16 subunits and, using C-terminal microtubule-binding domains (34, 74), tether SUMOylated hSpt16 to microtubules to prevent FACT-dependent ETS-1 expression in the nucleus (74) (Fig. 1). Unlike wild-type VACV, strains lacking A51R strongly induced ETS-1 expression during infection and displayed attenuated replication in cell culture that could be rescued by hSpt16 RNA interference (RNAi)-mediated depletion (74, 80). Electron microscopy studies of VACV A51R knockout virus-infected cells suggested that the FEAR pathway restricts VACV at the stage of virion morphogenesis, as these mutant viruses produced reduced numbers of mature VACV particles, and this phenotype could be reversed by depletion of hSpt16 levels (74). Furthermore, mice inoculated with VACV strains lacking A51R or encoding A51R mutants that cannot bind SUMOylated hSpt16 displayed increased survival rates compared to mice infected with wild-type VACV, suggesting FEAR antagonism promotes poxvirus pathogenesis (34, 74, 80).

Recently, RNA virus-encoded inhibitors of the FEAR pathway have also been discovered, but these appear to function through mechanisms distinct from poxvirus A51R proteins. For instance, the matrix (M) protein of VSV promotes proteasome-dependent degradation of SUMOylated hSpt16 and prevents ETS-1 nuclear translocation in human cells (73). VSV strains encoding M protein mutants that cannot degrade SUMOylated hSpt16 strongly induce ETS-1 expression during infection and display attenuated replication in human cells (73). Yet, these replication defects were rescued by knockdown of either hSpt16 or ETS-1 (73). Curiously, unrelated paramyxoviruses also promote the proteasomal degradation of SUMOylated hSpt16. SeV and HPIV-1 both encode immunomodulatory C proteins that are ~70% identical to one another but show no sequence or structural similarity to VSV M proteins (73). However, as with VSV M, the expression of C proteins in the absence of infection was sufficient to promote degradation of SUMOylated hSpt16 proteins (73). Remarkably, the expression of a 23 a.a. N-terminal motif from paramyxovirus C proteins was also sufficient to degrade intracellular SUMOylated hSpt16 levels (73). Structural predictions indicated that this motif falls within an alpha helix conserved between SeV and HPIV-1 C proteins and thus may be important for promoting SUMOylated hSpt16 depletion (73). Notably, the C-terminus of paramyxovirus C proteins mediates interaction with STAT1 (81). Thus, paramyxovirus C proteins use N-terminal domains for FEAR pathway inhibition, while their C-terminal domains antagonize IFN-I signaling.

FACT and ETS-1 upregulation are associated with oncogenesis, transformation, and tumor invasion (82–85), leading to the development of small molecule FACT inhibitors called “curaxins” currently in clinical trials to treat human malignancies (86). Coincidentally, VSV mutant strains are similarly being pursued as an oncolytic agent in the clinic. However, some cancer cell types are refractory to oncolytic VSV strain infection, and the FEAR pathway has been recently implicated in the restriction of oncolytic VSV strains in some of these cancer cell types (73, 87–89). Curaxin treatment together with oncolytic VSV infection increased cancer cell death in multiple resistant cell types, suggesting that FEAR inhibition through curaxin treatment can sensitize refractory cancer cells to oncolytic VSV infection (73). Thus, this combinatorial therapeutic approach may broaden the use of oncolytic VSV therapy against cancer cell types that would otherwise be refractory to such virotherapy.

Interestingly, FACT has been reported to have additional roles outside of the FEAR pathway during infection that can either help or hinder viral replication. For example, RNAi screens identified both hSpt16 and SSRP1 subunits as suppressors of transcription by integrated retroviruses, such as human immunodeficiency virus-1 (HIV-1; *Retroviridae*) (90). In contrast, FACT is recruited by members of the *Herpesviridae* family to promote viral replication. The herpes simplex virus-1 (HSV-1; *Herpesviridae*)-encoded ICP22 protein was found to interact with both FACT subunits, which resulted in the redistribution of FACT to viral genomes, facilitation of viral transcription, and suppression of FACT-dependent cellular gene transcription (91, 92). HCMV was also shown to recruit FACT to drive viral reactivation by helping to activate the major immediate early promoter, which controls the expression of immediate early genes encoding viral transactivators required for lytic replication (93). Curaxins reduced HCMV reactivation and lytic infection, suggesting that such drug treatments may be capable of maintaining HCMV latency (93–95). Together, these observations, along with the conservation of FACT from yeast to humans, suggest that the FACT complex has likely played many diverse roles in the regulation of viral replication throughout eukaryotic evolution. Moreover, FACT inhibitor drugs may be useful therapeutics for either promoting the replication of oncolytic viruses antagonized by FACT (i.e., to improve oncolytic virotherapy efficacy) or for inhibiting the active replication of viruses that require FACT for their life cycle, as in the case of herpesviruses.

### Degradation of virus particles and components: autophagy in antiviral defense

**Fig 2:**
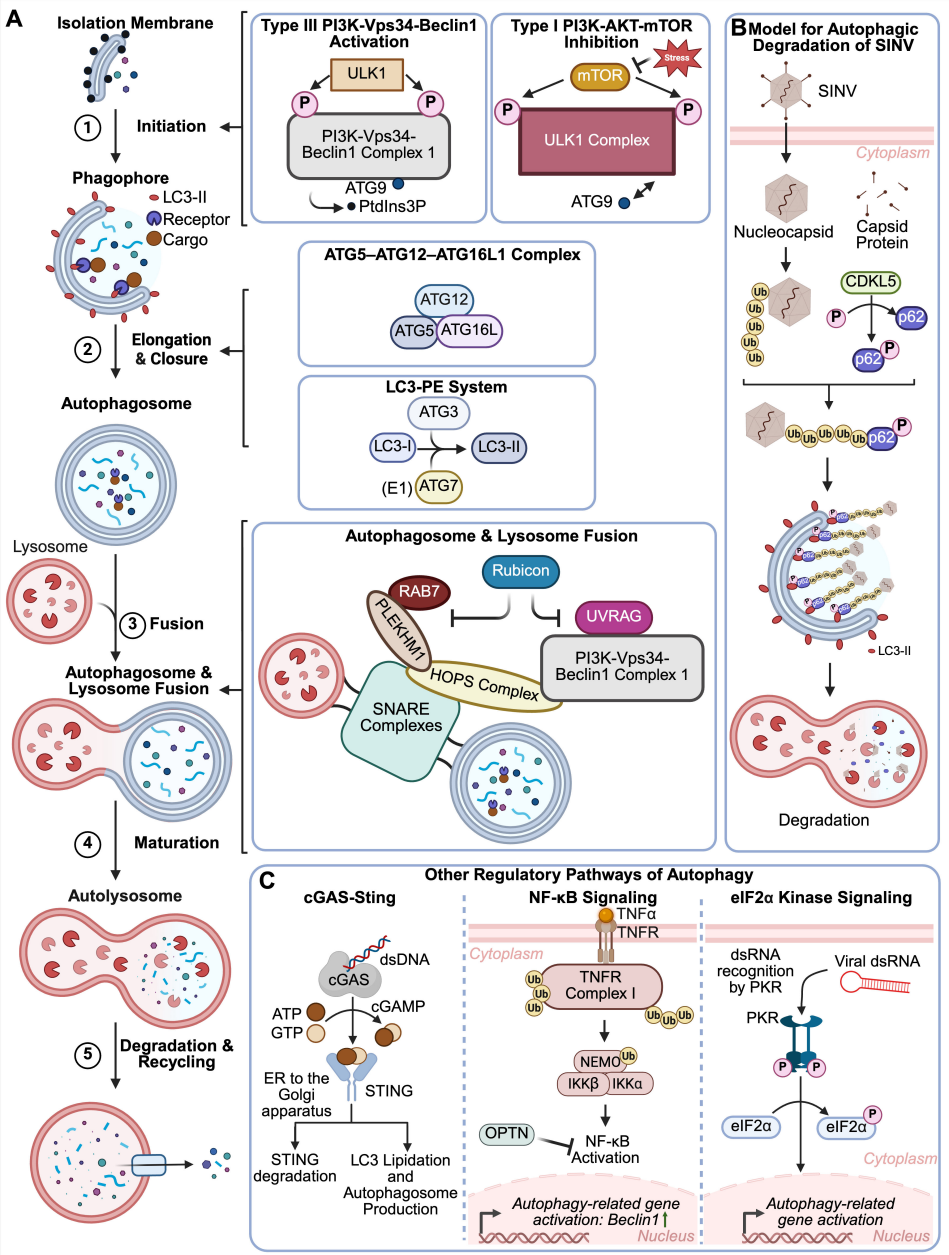
Autophagy and antiviral defense. Adapted from references 98 and 119. (**A**) The steps of autophagy (Step 1: initiation; Step 2: phagophore elongation and closure to form autophagosome; Steps 3 and 4: autophagosome-lysosome fusion and formation of autolysosome; Step 5: degradation and recycling of autolysosome contents) are illustrated on the left, accompanied by depictions of the key proteins involved. (**A**, Step 1) The autophagy pathway is regulated by the PI3K/AKT/mTOR cellular signaling pathway, which influences the activity of the ULK1 complex. ULK1 activates the PI3K-Vps34-Beclin1 complex 1, initiating phagophore formation. (**A**, Step 2) At this step, host cargo receptors tether cargo to the membrane by interacting with LC3, targeting cargo for degradation. The ATG5–ATG12–ATG16L system and LC3-PE system aid in membrane elongation. (**A**, Steps 3 and 4) Proteins, including Rab GTPases, SNAREs, and the HOPS complex, coordinate the autophagosome-lysosome fusion process, leading to mature autolysosome formation (**A**, Steps 3 and 4). This facilitates the enzymatic breakdown and recycling of autolysosome contents (**A**, Step 5). (**B**) Model for how virophagy targets SINV for degradation in cells via the action of CDKL5 (119). (**C**) cGAS-STING (left), NF-κB (middle), and eIF2α kinase (right) signaling pathways can also regulate autophagy. (**C**, left) In the cGAS-STING pathway, STING is activated by cGAMP, after which it moves from the ER to the Golgi apparatus. STING can help promote LC3 lipidation, supporting a necessary step in autophagy. STING itself is also targeted for autophagic degradation, which ensures regulation of the pathway. (**C**, middle) NF-κB inhibition increases the expression of autophagy-related genes, while downregulating p-mTOR and p-AKT. This inhibition is partly due to the host receptor, OPTN, which outcompetes NEMO for binding and thereby blocks NF*-*κB activation. (**C**, right) In the PKR signaling pathway, PKR senses viral dsRNA, triggering the phosphorylation of the eIF2α translation initiation factor and activation of autophagy-related genes. Figure created with BioRender.com

Autophagy is a highly conserved catabolic pathway in eukaryotic cells that supports cellular homeostasis by degrading and recycling superfluous or damaged organelles, proteins, lipids, and nucleic acids through the use of lysosomes (77–80). When cells experience stressors, such as pathogen invasion, autophagy aids in the maintenance of cellular integrity and clearance of pathogens through the action of autophagosomes, which are double-membrane vesicles that deliver cellular components to lysosomes for degradation (96–99). Over 30 autophagy-related (ATG) proteins orchestrate the different steps of autophagy, including initiation, phagophore membrane elongation and closure, autophagosome maturation and fusion with lysosomes, and degradation and recycling (Fig. 2A, Steps 1–5) (100, 101). Autophagy initiation can be activated through inhibition of the PI3K-AKT-mTOR signaling pathway or activation of the PI3K-VPS34-Beclin1 pathway (Fig. 2A, Step 1) (98, 102–104). Initiation involves the ULK1 complex activation of the type III phosphoinositide 3-kinase (PI3K) complex I (including Beclin1 and Vps34), generating phosphatidylinositol-3-phosphate (PtdIns3P) on membranes and forming the phagophore (Fig. 2A, Step 1) (105). Elongation and closure of the phagophore to form the autophagosome are supported by both the ATG5–ATG12–ATG16L1 complex, an E3-like ligase, promoting LC3 oligomerization on the phagophore membrane and the LC3-phosphatidylethanolamine system converting LC3 to LC3-II (Fig. 2A, Step 2) (106–110). LC3-II is attached to membranes during elongation, serving as docking sites for host receptors that deliver interaction-specific cargo (Fig. 2A, Step 2) (111, 112). Subsequent fusion of autophagosomes with lysosomes to generate mature autolysosomes involves the coordination of many proteins, including SNARE complexes, Rab GTPases, and HOPS, which facilitate membrane apposition (Fig. 2A, Steps 3 and 4) (112–116). Rab7 and UVRAG promote fusion (112–115), while Rubicon negatively regulates autophagosome maturation (Fig. 2A, Steps 3 and 4) (117, 118). These autolysosomes then degrade their contents with hydrolases, and degradation products are subsequently recycled (Fig. 2A, Step 5) (117, 118).

Autophagy plays a crucial role in antiviral defense by degrading viral components, stimulating immune responses, and modulating inflammation (97–99, 120). Through a process called “virophagy,” autophagy selectively targets viral particles and components, enclosing them in autophagosomes for degradation via lysosomes (119, 121, 122). Host selective autophagy receptor proteins, such as p62/SQSTM1, TAX1BP1, NDP52/CALCOCO2, NBR1, and OPTN, can recognize specific viral cargo, tethering them to the phagophore through LC3-interacting regions and ubiquitin-binding domains (Fig. 2B) (97, 123–125). One early discovered example of virophagy involves p62-directed degradation of the Sindbis virus (SINV; *Togaviridae*) capsid in the autophagosome (126, 127). More recently, host cyclin-dependent kinase-like 5 (CDKL5) was shown to promote p62 phosphorylation, which promoted interaction with the SINV capsid (Fig. 2B) (119). CDKL5 knockout mice exhibited increased viral antigen levels and neuronal cell death after SINV infection (119). Furthermore, challenges with several neurotropic viruses resulted in reduced survival rates in these knockout mice, suggesting CDKL5-mediated virophagy is a critical antiviral defense *in vivo* (119). p62 is also involved in targeting VP1 and VP3 of Seneca Valley virus (*Picornaviridae*) (128), HSV-1 (129), and capsid proteins of Chikungunya virus (*Togaviridae*) (130). Besides ubiquitin tags, galectin decoration of viral capsid proteins can also facilitate p62-mediated virophagy (131, 132). Another cargo receptor, OPTN, targets HSV-1 proteins for degradation in the central nervous system (133). A diverse array of additional host factors can function as cargo receptors, which may explain how host cells can utilize autophagy to recognize and eliminate disparate viral pathogens (134–138).

In addition to canonical IFN pathways (120, 139–142), cGAS-STING (143, 144), NF-κB (145–147), and eIF2α kinase signaling (141, 148, 149) pathways influence autophagy activation in an IFN-I-independent manner (Fig. 2C). The cGAS-STING pathway, known to recognize cytosolic DNA and trigger IFN production (150), has two recent reports suggesting STING has IFN-I-independent antiviral functions (Fig. 2C, left) (143, 144). In the presence of cGAMP, poly(dA:dT), or HSV-1 infection, STING activated a non-canonical autophagy response that is dependent on ATG5 but independent of canonical autophagy factors, such as ULK1 and p62 (Fig. 2C, left) (151, 152). Once activated, STING supported LC3 lipidation, which is essential for autophagy, through WIPI2 and ATG5 (Fig. 2C, left) (152). The pathway is also tightly regulated by selective autophagy; p62 targets ubiquitinated cGAS and STING for degradation, preventing overactivation of the pathway (Fig. 2C, left) (153). Given that cGAS-STING signaling is conserved in some invertebrates (which typically lack IFNs), the activation of autophagy may be the primordial function of the cGAS-STING signaling pathway, predating its role in activating IFN-I signaling that arose during the evolution of vertebrates (152). Interestingly, TRIM23, an E3 ubiquitin ligase and GTPase, was recently found to be crucial for a cGAS-STING-TBK1-TRIM23 antiviral autophagy pathway (154). HSV-1 infection or cGAS-STING stimulation was found to induce TBK1-mediated TRIM23 phosphorylation, which, in turn, triggered TRIM23 autoubiquitination and GTPase activity, leading to autophagy and suppression of HSV-1 replication (154).

Cross-talk between autophagy and other immunity pathways can also regulate autophagic antiviral responses (Fig. 2C). For example, inhibition of NF-κB pathway signaling can promote autophagy through upregulation of ATG protein expression (Beclin1) and induction of autophagosome formation; this inhibition also reduced p-AKT and p-mTOR levels, further driving autophagy activation (Fig. 2C, middle) (145–147). During infection, OPTN not only helped target ubiquitinated cargo for degradation but also inhibited NF-κB activation to promote autophagy (Fig. 2C, middle) (145, 155). eIF2α kinase activation by various stress stimuli, including starvation and viral infection, leads to eIF2α phosphorylation and also induces ATG gene expression (Fig. 2C, right) (148). During HSV-1 infection, the dsRNA-sensing eIF2α kinase PKR phosphorylates eIF2α and induces antiviral autophagy (Fig. 2C, right) (141). Interestingly, during infection with pseudorabies virus (*Herpesviridae*), the host USP14 deubiquitinase becomes inactivated, which serves to activate the PERK eIF2α kinase and the unfolded protein response (156). Together, this promotes the degradation of the viral transcriptional regulator VP16 via p62-mediated autophagy, thereby suppressing viral replication (156).

The importance of autophagy in antiviral defense is underscored by the discovery of many virally encoded inhibitors of autophagy. Viruses often manipulate the PI3K-AKT-mTOR or PI3K-VPS34-Beclin1 pathways to exploit autophagic membranes or machinery while also avoiding degradation to benefit their replication (99, 104, 120). These viral antagonists employ diverse strategies to block autophagy-mediated degradation, ranging from the direct inhibition or degradation of ATG proteins to the prevention of lysosomal acidification (Table 2) (97, 129, 142, 157–187).

**TABLE 2:** Viral inhibitors of host protein targets in antiviral autophagy pathways

Viral family	Virus	Viral proteins	Putative mechanism	Reference
*Herpesviridae*	Herpes simplex virus 1	ICP34.5	Interaction with Beclin-1 inhibits the PI3K-Vps34-Beclin1 complex	(142)
		US3	mTORC activation, which inhibits ULK1 activity and Beclin1	(157)
		US11	Decreases p62 ubiquitination	(129)
	Human cytomegalovirus	TRS1	Interaction with Beclin1 inhibits initiation through the PI3K-Beclin1-ATG14 complex	(158, 159)
		IRS1	Interaction with Beclin1 inhibits initiation through the PI3K-Beclin1-ATG14 complex	(158, 159)
	Epstein-Barr virus	BFRF1	Decreases expression of Rab7	(160)
	Kaposi’s sarcoma-associated herpesvirus	v-FLIP	Interaction with ATG3 prevents processing of LC3	(161, 162)
		v-GPCR	Activates mTOR; downregulates ATG14L expression	(163, 164)
*Picornaviridae*	Picornaviruses	3C	Degradation of Atg5/Atg12	(165)
	Coxsackievirus B3	2A	Cleaves p62	(166)
		3C	SNAP29 and PLEKHM1 cleavage to disrupt SNARE complex	(167)
	Enterovirus-D68	3C	Cleaves p62	(188)
			SNAP29 and PLEKHM1 cleavage to disrupt SNARE complex	(188)
	Foot-and-mouth disease virus	3C	Interaction with Beclin1 prevents autophagosome and lysosome fusion	(189)
*Orthomyxoviridae*	Influenza A virus	M2	Interaction with Beclin1 prevents autophagosome and lysosome fusion	(168, 169)
*Hepadnaviridae*	Hepatitis B virus	HBx	Impairment of lysosomal acidification	(170)
*Retroviridae*	Human immunodeficiency virus-1	Nef	Employs PRKN and BCL2 as an inhibitor of Beclin1 to impair initiation	(171)
		E	Activation of mTOR	(172)
*Flaviviridae*	Zika virus	NS2B3	Cleaves p62	(173, 174)
	Hepatitis C virus	NS4B	Induces the expression of Rubicon and UVRAG, manipulating autophagy for viral replication	(190)
*Asfarviridae*	African swine fever virus	A179L	vBcl-2; interacts with Beclin-1, inhibiting the PI3K-Vps34-Beclin1 complex	(191)
*Coronaviridae*	Severe acute respiratory syndrome coronavirus 2	ORF3a/ ORF7a	Sequesters VPS39/UVRAG, preventing SNARE assembly	(97, 174, 177–187)
	Severe acute respiratory syndrome coronavirus			
	Middle East respiratory syndrome coronavirus			

Aberrant regulation of autophagy has been tied to various pathological conditions, including neurodegenerative diseases (192–196), cancers (197, 198), inflammation (199), and infections (200, 201). Importantly, drugs targeting autophagy have shown therapeutic potential in a wide array of viral infections using cell culture and mouse model systems (122). These include autophagy activators, rapamycin (202–204), metformin (205, 206), and CSC27 (207), as well as autophagy inhibitors, wortmannin (208) and corticosteroids (209–212). Clearly, there may be utility in developing therapeutic strategies to manipulate autophagic pathways for the treatment of viral disease.

### Stem cells and the endogenous RTase/RNase H-mediated antiviral system

Even though IFN-I responses are often the most critical antiviral defense induced during viral infection of most differentiated cell types, pluripotent stem cells do not elicit a productive IFN response after infection with viruses, exposure to PAMPs, or when treated with recombinant IFN (13, 213). Interestingly, evidence suggests that the maintenance of stem cell pluripotency is compromised when these cells are engineered to express a constitutively active form of IRF7 (which induces IFN-I responses) (13). These findings suggest that the IFN-I response and stem cell pluripotency may be incompatible with one another, possibly explaining why stem cells do not employ the IFN-I response for antiviral defense (13). However, this surprising finding has led to the suggestion that stem cells may either be resistant to viral infections because they lack expression of receptors or co-receptors required for viral entry (214), or because these cells utilize alternative, IFN-I-independent antiviral responses to block infection. For example, antiviral RNAi responses have recently been reported to restrict ZIKV and SARS-CoV-2 replication in mammalian stem cells through the action of a shortened Dicer isoform termed antiviral Dicer, which is particularly active in dicing long dsRNAs (215). However, the role of RNAi in mammalian antiviral defense remains controversial and has been recently reviewed elsewhere (28).

Recently, a nucleic acid-based antiviral system, distinct from RNAi, was identified in mouse embryonic stem cells (ESCs) that utilizes the action of endogenous reverse transcriptases (RTases) to mediate RNA virus restriction. Mammalian genomes contain a considerable number of residual endogenous retrovirus (ERV) sequences—an outcome of historical retroviral infection, reverse transcription, and integration events. While most ERVs are defective, some encode retroviral proteins, such as RTases, that are highly active in ESCs compared to differentiated, somatic cells (216–219). Mechanistically, when mouse ESCs are infected with RNA viruses, RTases function to generate viral DNA copies from viral RNA templates, creating viral DNA/RNA duplexes (219). This unique hybrid structure is recognized and cleaved by RNase H, which specifically degrades the RNA strand, ultimately leading to viral inhibition (219). This newly identified endogenous RTase/RNase H-mediated antiviral system (termed “ERASE” for short) helps to explain how stem cells can resist viral infection without inducing IFN-I responses.

Currently, there are no known viral inhibitors of ERASE. Although mouse ESCs pre-treated with azidothymidine (AZT), a mammalian RTase inhibitor, increased viral mRNA, protein, and titers of positive-sense ssRNA encephalomyocarditis virus (*Picornaviridae*) and mouse hepatitis virus (*Coronaviridae*) (219). However, HSV-1 titers were unaffected by AZT treatment (219), suggesting that endogenous mouse ESC RTases may exclusively restrict RNA virus replication. It is still unclear if ERASE may have activity against negative-sense ssRNA and dsRNA viruses, retroviruses, or cytoplasmically replicating DNA viruses. ERASE represents another striking example of how nucleic acid-based antiviral immunity mechanisms can contribute to IFN-I-independent defenses. Given the diversity of RTases and RNases encoded by mammals, additional ERV-derived proteins may possess similar functional capabilities that have yet to be discovered (220).

### Impeding viral release: septins

Septins are a conserved family of GTP-binding proteins that were initially identified as ~10 nm nonpolar filaments forming ring-like structures at the bud necks of *Saccharomyces cerevisiae* (221). Subsequent studies revealed that septins function at the plasma membrane to regulate a range of cellular processes, including cytokinesis, ciliogenesis, phagocytosis, and innate immunity (222, 223). Septins assemble into hetero-oligomeric complexes that can further polymerize into filaments, rings, and cage-like structures (224). These higher-order assemblies serve as scaffolds for intracellular protein localization and as barriers that compartmentalize membrane domains (223, 225). In humans, the 13 septin genes are grouped into four subfamilies based on sequence homology: SEPT2 (SEPT1, 2, 4, 5), SEPT3 (SEPT3, 9, 12), SEPT6 (SEPT6, 8, 10, 11, 14), and SEPT7 (SEPT7) (225–228). Members from each subfamily co-assemble into complexes capable of forming functional septin structures (223, 229, 230).

During bacterial infection, septins mediate cell-intrinsic immunity by forming filamentous cages that entrap motile pathogens, such as *Shigella flexneri* (231, 232). Sensing the micron-scale membrane curvature as *S. flexneri* invades cells and escapes from its phagosomal vacuole into the host cytoplasm, septins recognize the poles of growing *S. flexneri* and build filament cages around the bacterium during its early stages of replication (231, 232). This physical septin cage inhibits actin tail formation, a mechanism used by *S. flexneri* for intracellular propulsion, as well as targets the bacteria for degradation via autophagy (232, 233). Notably, septin entrapment is enhanced against growing bacteria, as non-replicating bacteria partially evaded cage formation (231, 234). The direct mechanisms behind this escape strategy and the evolutionary tradeoffs for having this escape mechanism have yet to be fully elucidated.

**Fig 3:**
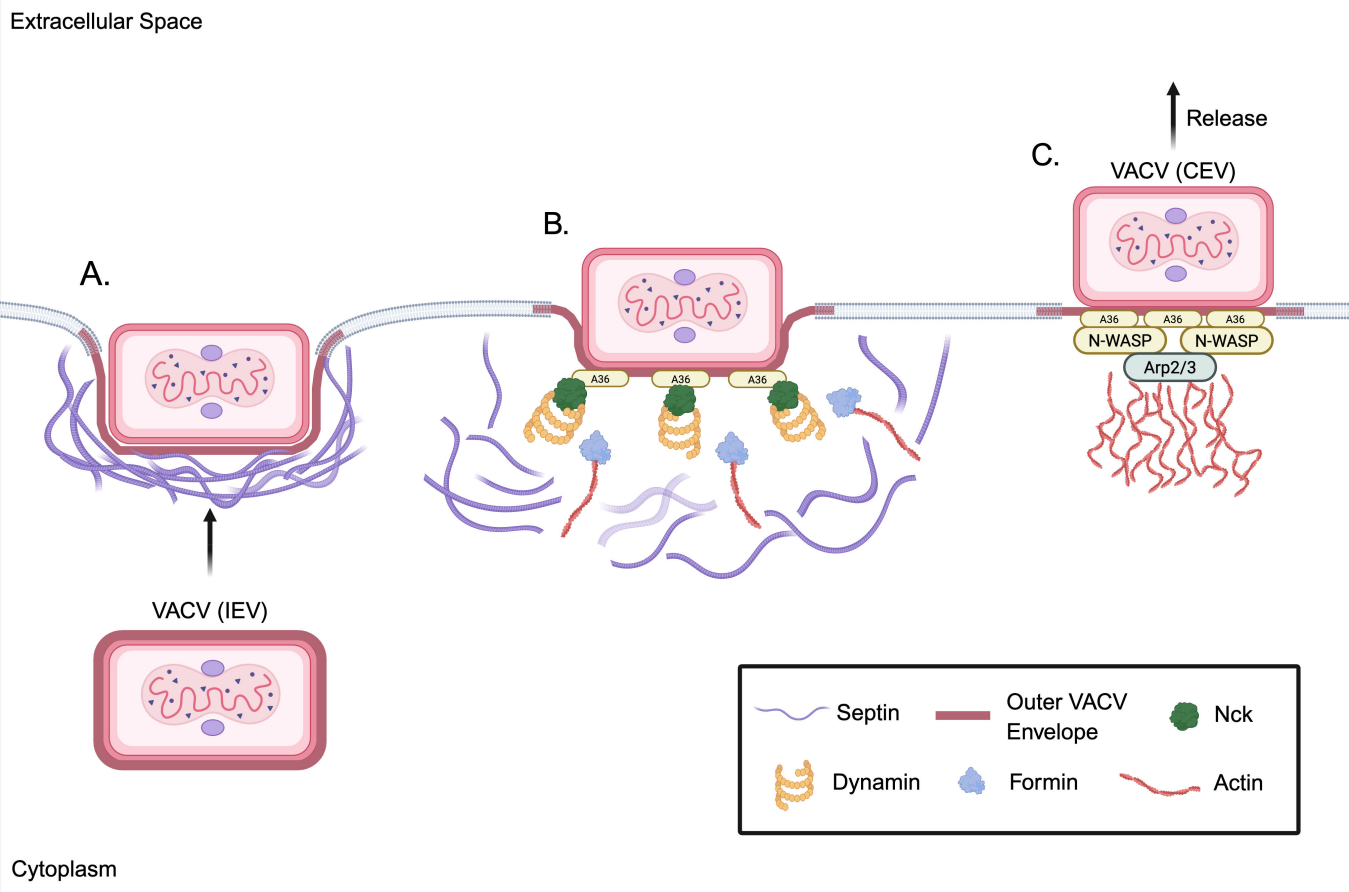
Impediment of VACV egress by septin entrapment. (**A**) Septin cage entrapment of VACV virion. Upon arrival near the plasma membrane, some intracellular enveloped virus (IEV) particles are recognized by host septins (purple), which assemble into filamentous cages (226, 240). These structures physically trap the virus as the outer viral envelope (dark pink layer) fuses with the plasma membrane, inhibiting actin motility and effectively halting VACV egress (238–240). (**B**) Initiation of viral escape and actin nucleation. The viral envelope protein A36R is phosphorylated, enabling the recruitment of the host adaptor protein Nck (green) (240). Nck, in turn, recruits dynamin (orange spirals), a GTPase that contributes to local septin disassembly (240). As septins are removed, formins (blue) are recruited, initiating the formation of linear actin filaments (red) beneath the virion (240). (**C**) Actin tail formation and outward propulsion. The viral particle then engages the Arp2/3 complex via N-WASP (240), leading to the formation of branched actin filaments. These actin tails push the virion away from the membrane, producing a cell-associated enveloped virus poised for transmission to neighboring cells (240). Figure adapted from reference 240 and created with BioRender.com.

The best-characterized antiviral roles of septins have been described in the context of VACV, which produces intracellular enveloped virions that egress from infected cells using both microtubule-dependent transport and actin-based motility (235–237). Septins were originally implicated in VACV restriction by two independent genome-wide RNAi screens, which identified several septins (e.g., SEPT7, SEPT11, etc.) as antiviral factors (238, 239). RNAi-mediated knockdown of septins resulted in enhanced VACV replication and increased plaque size, indicating that septins restrict VACV spread (240). Without septins, VACV cell-associated enveloped viruses (CEVs; i.e., virions that exit the cell but remain attached to the membrane where they facilitate Arp2/3 complex-dependent actin polymerization) (237, 241) exhibited more frequent and longer-lived actin tails, indicating that septins suppress actin tail formation required for VACV egress (240). Similar to their interactions with *S. flexneri,* septins were found to assemble into cage-like structures at the plasma membrane that trap CEVs (Fig. 3A) (226, 240). However, this restriction appears to be transient as septin cages are eventually dismantled by localized actin remodeling events facilitated by VACV A36R proteins that engage with the host SH2/SH3 domain-containing adaptor Nck (Fig. 3B) (240). Nck then recruits dynamin to promote septin disassembly, which is followed by formin-mediated actin polymerization and displacement of septin structures (Fig. 3B) (240). This subsequently permits actin tail formation and facilitates VACV egress (Fig. 3C) (240).

While VACV represents the primary model for studying septin-mediated antiviral defense, emerging evidence suggests potential interactions between septins and other viral pathogens. For example, the ZIKV-encoded NS2B3 protease was shown to target SEPT2 and cause delayed cytokinesis in neural stem cells (242). Although it is unclear how this SEPT2 targeting impacts the viral life cycle, it has been suggested that this may contribute to infection-associated neurotoxicity (242). Interestingly, although septins do not appear to be ISGs (243), there have been reports of differential regulation of septins at both the transcriptional and protein levels during viral infection (244). For example, infection with hepatitis C virus (HCV) was associated with transcriptional upregulation of septins that, in turn, underwent filament assembly to regulate lipid droplet growth (245), and SEPT2 and SEPT6 have been implicated in supporting HCV replication (245), suggesting that septins can play antiviral or proviral roles (244). In contrast to infection-induced septin filamentation observed with HCV, the human immunodeficiency virus Tat protein was shown to promote disruption of SEPT7 filaments (246). Thus, there are clearly complex relationships between septins and viral pathogens that remain poorly understood.

### Inhibition of viral replication and spread through programmed cell death

While cell intrinsic factors attempt to limit viral infection, PAMPs and damage-associated molecular patterns (DAMPs) activate programmed cell death pathways to restrict viral spread, representing a “last resort” innate response (247). Cell death occurs via non-lytic or lytic programs (248, 249). Apoptosis is non-lytic and immunologically silent, maintaining membrane integrity while inducing morphological changes, such as nuclear fragmentation and membrane blebbing, that signal clearance by phagocytic cells (250, 251). In contrast, pyroptosis and necroptosis are lytic and pro-inflammatory, wherein cellular membranes are ruptured and release cellular contents that trigger strong immune responses (252). Although programmed cell death mechanisms can be potent antiviral defenses, viruses have often evolved strategies to prolong viral replication or promote viral spread by inhibiting or usurping key components of these cell death pathways (253, 254). Table 3 provides examples of how viruses regulate three key antiviral programmed cell death pathways: apoptosis, pyroptosis, and necroptosis.

**TABLE 3:** Viral regulators of cell death programs

Viral family	Virus	Viral proteins	Putative mechanism	Reference
Apoptosis pathway				
*Poxviridae*	Vaccinia virus	F1L (OPG045)	vBcl-2, Bim inhibitor	(255–258)
		N1L (OPG035)	vBcl-2, Bad, tBid inhibitor	(259, 260)
		B22R (OPG208)	CASP8 inhibitor	(261, 262)
		B13R (OPG199)	CASP8 inhibitor	(263, 264)
		M1L (OPG037)	CASP9 processing inhibitor	(265)
	Variola virus	F1L (OPG045)	vBcl-2, BID, BAK, and BAX inhibitor	(266)
	Cowpox virus	CrmA (OPG199)	Caspase inhibitor	(267, 268)
	Myxoma virus	M11L	vBcl-2, interacts with BAK to inhibit BAX	(269–272)
	Molluscum contagiosum virus	MC159	FADD (CD95 FLICE recruiting adaptor protein) inhibitor	(273)
	Ectromelia virus	EVM025 (OPG045)	vBcl-2, interacts with BAK to inhibit BAX	(274)
	Sheeppox virus	SPPV14	vBcl-2, Bim, Puma, Bmf, and Hrk inhibitor	(275, 276)
	Deerpox virus	DPV022	vBcl-2	(277)
	Orfvirus	ORFV125	vBcl-2, Bax, Bak, Puma, and Hrk inhibitor	(278–280)
	Tanapox virus	TANV16L	vBcl-2, Bax, Bim, and Puma inhibitor	(281)
*Herpesviridae*	Murine γ-herpesviruses 68	M11	Fas- and TNF-induced inhibitor	(282)
	Murine cytomegalovirus	pM36	Pro-caspase-8 inhibitor	(283)
	Epstein-Barr virus	BHRF1	vBcl-2, BECN1/Beclin 1	(284, 285)
		BALF1	vBcl-2, BAX/BAK inhibitor	(284, 286)
	Kaposi’s sarcoma-associated herpesvirus	Ks-Bcl-2	vBcl-2, Aven (pro-survival, Apaf-1/CASP9 inhibitor) inhibitor	(287, 288)
	Herpesvirus saimiri	ORF16	vBcl-2, interacts with BAX/BAK. CASP3 inhibitor	(289, 290)
		ORF71	vBcl-2, FADD inhibitor	(291)
	Herpes simplex virus 1	ICP22	CASP3 inhibitor	(292)
	Equine herpesvirus-2	E8	Binds to pro-CASP8	(273, 291)
*Asfarviridae*	African swine fever virus	A179L	vBcl-2, PARP, CASP8, CASP3 inhibitor	(293–295)
*Adenoviridae*	Adenovirus	E1B19K	vBcl-2, BAX/BAK inhibitor	(296–299)
*Picornoviridae*	Coxsackievirus B4	2BC	CASP3 inhibitor	(300)
		2A	Apoptosis activator via Bid cleavage	(301)
		3C	Apoptosis activator via upregulation of BAX and Bid cleavage	(301)
*Matonaviridae*	Rubella virus	Capsid	BAX inhibitor	(302)
*Flaviviridae*	Zika virus	NS4	Apoptosis activator via Bax interaction	(303)
	Dengue virus	Capsid	Binds to calcium modulating cyclophilin-binding ligand, inhibiting CASP3 activation	(304)
	Langat virus	NS3	Cleaves CASP8 to initiate apoptosis	(305)
*Reoviridae*	Reovirus	μ1	Promotes CASP3 activation	(306, 307)
Pyroptosis pathway				
*Poxviridae*	Vaccinia virus	F1L (OPG045)	Bind and inhibit NLRP1 inflammasome	(308)
		B13R (OPG199)	Inhibit caspase-1-induced inflammasome activation	(309)
		A47L (OPG177)	Viral GSDMD, inflammatory pan-CASP inhibitor	(310)
	Myxoma virus	M013L	Viral PYD-only protein 1 decoy protein	(311)
	Shope Fibroma virus	S013L	Viral PYD-only protein 1 decoy protein	(311, 312)
*Herpesviridae*	Kaposi's sarcoma-associated herpesvirus	ORF63	NLRP1 inflammasome inhibitor	(313)
	Herpes simplex virus 1	ICP0	NLRP1 inflammasome inhibitor	(314)
	Murine cytomegalovirus	M84	AIM2 inflammasome inhibitor	(315)
*Coronaviridae*	Severe acute respiratory syndrome coronavirus 2	NP	Blocks GSDMD cleavage	(316)
		NSP5	Cleaves GSDMD blocking oligomerization	(317–319)
	Middle East respiratory syndrome coronavirus	NSP5	Cleaves GSDMD blocking oligomerization	(317)
*Picornaviridae*	Enterovirus-71	3Cpro	Cleaves GSDMD blocking oligomerization	(320, 321)
	Simian sapelovirus	3Cpro	Cleaves GSDMD blocking oligomerization	(320, 321)
*Papillomaviridae*	Human papillomavirus	E7	Ubiquitinates IFI16 inflammasome for degradation	(322)
*Paramyxoviridae*	Measles virus	V	Interacts with NLRP3 to block oligomerization, inhibiting IL-1β secretion	(323)
	Sendai virus	V		(324)
	Nipah virus	V		(324)
	Human parainfluenza virus 2	V		(324)
*Orthomyxoviridae*	Influenza A virus	M2		(325)
		NS1	Inhibits ACS ubiquitination, suppressing NLRP3 inflammasome activation	(326)
			Interacts with NLRP3, inhibiting activation	(325, 327)
Necroptosis pathway				
*Orthomyxoviridae*	Influenza A virus	NS1	Promotes MLKL oligomerization	(328)
*Poxviridae*	Vaccinia virus	E3L (OPG065)	Sequesters zRNA, inhibiting PAMP detection	(329)
	Variola virus	E3L (OPG065)	Sequesters zRNA, inhibiting PAMP detection	(329)
		G1R	Promotes RIPK3 ubiquitination for degradation	(330)
	Cowpox virus	CPXV006	Promotes RIPK3 ubiquitination for degradation	(330)
	Mpox virus	J1R	Promotes RIPK3 ubiquitination for degradation	(330)
	Ectromelia virus	EVM002	Promotes RIPK3 ubiquitination for degradation	(330)
	Swinepox virus	E3L	Sequesters zRNA, inhibiting PAMP detection	(329)
		vMLKL, SPV140	MLKL decoy protein, sequestering RIPK1 and -3	(331)
	Sheeppox virus	E3L	Sequesters zRNA, inhibiting PAMP detection	(329)
	Goatpox virus			
	Lumpy skin disease virus			
	Cotiapox virus	vMLKL, COTV157	MLKL decoy protein, sequestering RIPK1 and -3	(331)
	BeAn virus	vMLKL, BAV Rmil		
	Myxoma virus	vMLKL, M147R		
*Herpesviridae*	Murine cytomegalovirus	M45	RIPK3 inhibition	(332, 333)
	Human cytomegalovirus	UL36	Promotes MLKL degradation	(334, 335)
		IE1	Promotes RIPK3 ubiquitination	(336)
	Herpes simplex virus 1	ICP6	Decoy protein inhibiting RIPK1/RIPK3 phosphorylation	(332, 337, 338)
	Herpes simplex virus 2	ICP10	Decoy protein inhibiting RIPK1/RIPK3 phosphorylation	(337)
	Epstein-Barr virus	LMP1	RIPK1 ubiquitination	(339)
			Hypermethylation of RIPK3 promoter	(339)
	Human papillomavirus	E6/E7	Downregulates RIPK3 expression	(340)
	Pseudorabies virus	VP22	Interacts with ZBP1, inhibiting RIPK3	(341)
*Paramyxoviridae*	Sendai virus	Y1/Y2	Downregulating cIAP1 expression to promote necroptosis	(342)
*Reoviridae*	Rotavirus	NSP4	Induces RIPK1/3, promoting viral spread	(343)

Apoptosis is mediated by intrinsic (mitochondrial) or extrinsic (death receptor) pathways, both converging to activate a cascade of cysteine aspartyl proteases, or caspases (CASPs), which cleave host substrates, resulting in cellular degradation and death (344, 345). Apoptotic initiation typically involves (i) extracellular ligand-receptor binding, including TNF family of ligands such as TNF-α, TRAIL, and FasL to TNFR1, DR4 or 5, and FasR, respectively; (ii) virus-host receptor interaction during attachment prior to entry; (iii) host sensing of viral replication intermediates; or (iv) virus-induced activation of the unfolded protein response (254, 346, 347).

**Fig 4:**
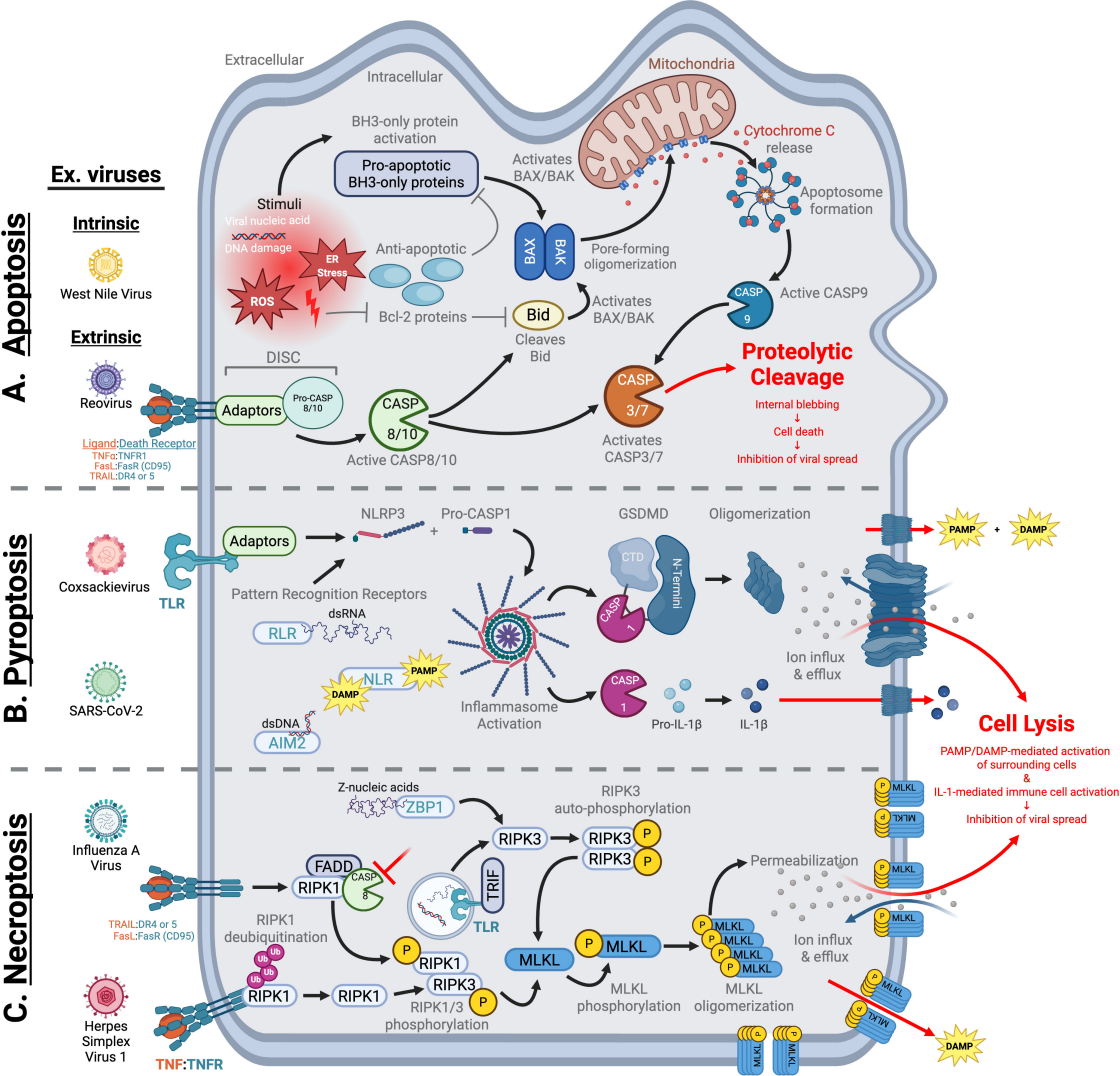
Cell death programs. (**A**) Apoptosis: virus-induced intrinsic cellular stress stimuli, such as hypoxia, reactive oxygen species, and DNA damage, relieve the inhibition of pro-apoptotic BH3-only proteins (e.g., Bid) by anti-apoptotic Bcl-2-like proteins, resulting in the pore-forming BAX/BAK complex to oligomerize and permeabilize the mitochondria. Loss of mitochondrial membrane integrity leads to the release of cytochrome C, which promotes apoptosome formation, activating effector CASP9. Extrinsic apoptosis is triggered upon extracellular ligands, such as TRAIL, FasL, and TNF-α, binding to their respective death receptors (DR4/5, FasR, and TNFR1). Intracellular adaptor proteins recruit pro-CASP8 and -10, forming DISC. Active effector CASP8 and -10 then cleave Bid, which can also activate BAX/BAK, feeding into the intrinsic pathway. Both intrinsic and extrinsic apoptosis rely on effector caspases to activate executioners CASP3 and 7, resulting in proteolytic cleavage of cellular targets. Degradation of nucleic acids, proteins, and membranes causes internal blebbing and cell death, inhibiting viral spread. (**B**) Pyroptosis initiates when PRRs (e.g., Toll-like receptors [TLRs], RLRs, NOD-like receptors [NLRs], and AIM2) are activated via ligand binding, which results in inflammasome complex assembly, where activated CASP1 cleaves GSDMD to relieve the N-terminal fragment from its autoinhibitory C-terminal domain (CTD). N-terminal GSDMD oligomerizes, perforating the cellular membrane, allowing intracellular PAMPs and DAMPs to be released from the cell, which activate surrounding cells. GSDMD pores also cause an ion influx into the cell, making the cell susceptible to osmotic lysis. CASP1 cleaves pro-IL-1β into mature IL-1β, which also escapes through GSDMD pores to initiate immune cell activation. (**C**) Necroptosis is initiated when CASP8 is inhibited during apoptosis. RIPK3 is either auto-phosphorylated or phosphorylated by RIPK1, where it then phosphorylates MLKL, triggering MLKL oligomerization to form a pore at the cellular membrane. TLR- or ZBP1-mediated sensing of viral nucleic acids promotes activation of necroptosis during virus infection. An ion influx into the cell also ensues, leading to osmotic pressure and membrane rupture. Examples of viruses that activate corresponding cell death pathways are shown on the left. Figure was created with BioRender.com.

The intrinsic pathway responds to intracellular physiological stresses that increase mitochondrial membrane permeability (348). Normally, B-cell lymphoma-2 (Bcl-2)-like proteins inhibit mitochondrial pore-forming Bcl-2-associated X protein (BAX) and Bcl-2 homologous antagonist/killer (BAK) from oligomerizing. However, oligomerization occurs when transcriptional or post-translational stress signals release Bcl-2-like proteins from BAX/BAK, or when pro-apoptotic BH3-only proteins directly activate BAX/BAK, causing mitochondrial outer membrane permeabilization and the release of apoptogenic molecules, like Cytochrome C (349–352). Cytochrome C unleashes active CASP9 to initiate CASP3 and CASP7 proteolytic activity (353–357). The extrinsic pathway is triggered when transmembrane death receptors bind to extracellular ligands, inducing oligomerization and formation of the death-inducing signaling complexes (DISC) through death domain interactions (358–362). DISC activates CASP8, which simultaneously cleaves pro-apoptotic Bid protein that feeds into the intrinsic pathway by activating BAX/BAK (363), and initiates CASP3 and CASP7 activation (Fig. 4A) (364). These executioner caspases cleave cellular components, exposing intracellular phospholipid phosphatidylserine on the cell surface, signaling for phagocytic clearance without provoking inflammation (365–367). Overall, apoptosis is a key antiviral defense, eliminating infected cells while minimizing immune activation.

Viruses have evolved an array of strategies to regulate apoptosis (Table 3). For example, many viruses encode viral Bcl-2-like proteins, which are structurally homologous to cellular Bcl-2 proteins (368). Herpesvirus saimiri (*Herpesviridae*) proteins (ORF16) and adenovirus (*Adenoviridae*) (E1B19K) express Bcl-2 homologs that inhibit BAX/BAK-mediated mitochondrial permeabilization (284, 285, 296). Poxviruses encode multiple Bcl-2 proteins that inhibit apoptosis at several junctures. For example, VACV F1 proteins prevent BAX/BAK oligomerization and inhibit CASP9 function, while VACV B13 suppresses initiator caspases in both intrinsic (CASP8) and extrinsic (CASP9) pathways (261, 263, 369–371). In contrast, reovirus (*Reoviridae*) μ1 proteins promote apoptosis to enhance viral spread while avoiding triggering an inflammatory response (306, 307). West Nile virus (WNV, *Flaviviridae*) NS3 proteins also induce CASP3-triggered apoptosis in neuronal cells, resulting in severe neuropathogenesis (372, 373). However, synthetic tetrapeptide aldehyde molecules that reversibly inhibit CASP3 and CASP7 specifically have promising therapeutic potential, as they significantly reduced WNV viral titers, as well as WNV-induced meningitis and encephalitis in murine brain tissues (372, 373). These diverse viral strategies underscore the central role of apoptosis in host antiviral defense.

Pyroptosis is a lytic programmed cell death pathway also driven by CASP activation but results in membrane rupture and inflammation (374–376). PRRs, like Toll-like receptors (TLRs) (377), AIM2 (378), RIG-I-like receptors (379), and NOD-like receptors (NLRs) (380), engage with PAMPs (e.g., viral nucleic acids [381–383]) or endogenously generated DAMPs (oxidized lipids [384], uric acid [385], reactive oxygen species [386], ATPs [387], etc.) during infection (388–390). PRR-ligand interactions result in the recruitment of adaptor proteins that organize a cytosolic multiprotein inflammasome complex, where pro-caspases are hydrolyzed into mature products to cleave gasdermin D (GSDMD)—the executioner protein (293, 391–393). The N-terminal fragment of GSDMD oligomerizes to non-selectively perforate the cell membrane, causing osmotic swelling and lysis (394–396). Concurrently, CASP1 processes pro-inflammatory cytokines pro-IL-1β and pro-IL-18 into their active forms (IL-1β and IL-18), which leak through the GSDMD pore to signal neighboring cells to initiate immune defenses and activate adaptive responses (397–400) (Fig. 4B). Thus, pyroptosis eliminates infected cells and triggers a strong inflammatory response to further limit pathogen spread.

As with apoptosis, viruses have evolved diverse mechanisms to disrupt inflammasome function to prevent cytokine production and cell death (Table 3). Orthopoxviruses, such as Mpox virus and variola virus, express serpin protease inhibitors that antagonize CASP1, preventing pyroptosis and IL-1β maturation (267, 401, 402). HSV-1 ICP0 proteins target NLRP1 and function to stabilize the inhibitory N-terminal NLRP1 fragment in a manner that depends upon the cytoplasmic localization and E3 ubiquitin ligase activity of ICP0 (314, 403). Coronaviruses, such as SARS-CoV-2, encode two factors that directly impede pyroptosis: the nucleocapsid protein binds to GSDMD, preventing CASP1-mediated GSDMD N-terminal processing, while NSP5 cleaves GSDMD to remove critical pore-forming N-terminal residues, preventing pyroptotic initiation (316, 404, 405). In contrast, SARS-CoV-2 proteins ORF3a, E, and M trigger NLRP3 inflammasome-activated pyroptosis in certain cell types, such as macrophages and pulmonary epithelial cells (406). Endothelial cells infected with picornaviruses, such as coxsackievirus and enterovirus A71, undergo NLRP3-mediated pyroptosis that elevates inflammatory responses. However, NLRP3 inhibitor (MCC950 sodium) treatment improved cell viability, reduced CASP1 activity, and attenuated IL-1β cytokine production during infection, suggesting that NLRP3 inhibitors may be a promising therapy against pyroptotic inflammation (407, 408). Together, these viral mechanisms aimed to subvert cellular pyroptosis demonstrate the critical role of inflammasome signaling in antiviral immunity.

Necroptosis is a caspase-independent, pro-inflammatory cell death program that morphologically resembles pyroptosis and serves as a fail-safe when apoptotic caspase activation is inhibited during infection (409–411). Necroptosis shares many initiating stimuli with apoptosis, for example, the TNF family of ligands, endoplasmic stress, and viral nucleic acids (412). A common feature of necroptotic receptors is that they contain a receptor-interacting protein kinase (RIPK) homology interaction motif (RHIM) that recruits receptor-interacting protein kinase 3 (RIPK3), activating mixed lineage kinase domain-like (MLKL) proteins (412–414).

TNF-α is the most well-characterized necroptotic trigger. When TNF-α is bound to its receptor, TNFR1, multiple proteins, including receptor-interacting protein kinase 1 (RIPK1), dock to the cytosolically protruding TNFR1 interface to assemble complex I (415–417). RIPK1 ubiquitination at complex I promotes cell survival, whereas deubiquitinated RIPK1 causes RIPK3 phosphorylation (418–421). Alternatively, Z-DNA binding protein 1 (ZBP1) is an intracellular PRR, sensing Z-form nucleic acids generated during viral infection, which operates independently of RIPK1 to trigger necroptosis (422–424). ZBP1 detection of these nucleic acid forms allows its RHIM domain to efficiently recruit and directly interact with RIPK3, resulting in RIPK3 autophosphorylation. TLR engagement with viral PAMPs also results in RIPK3 activation (250, 251). Regardless of RIPK1 involvement, active RIPK3 phosphorylates MLKL, induces a conformational change, and allows pore-forming oligomeric MLKL complexes to translocate to the cell membrane (425, 426). Although the precise mechanism and structural requirements governing MLKL assembly remain unknown, MLKL permeabilizes the cell membrane, resulting in NF-κB and mitogen-activated protein kinase signaling and decreased membrane integrity, causing a Ca^2+^ ion influx, promoting cell swelling and membrane rupture (Fig. 4C) (427, 428). Thus, necroptosis represents a vital backup cell death pathway, providing a robust immune response against pathogens upon apoptotic inhibition.

Though necroptosis likely evolved as a “back-up” cell death program, many viruses manipulate this pathway (Table 3). Epstein-Barr virus (EBV)-encoded latent membrane protein 1 suppresses necroptosis by interfering with RIPK1 ubiquitination, limiting the recruitment/assembly of complex I proteins and downstream RIPK3 phosphorylation, thereby maintaining cell survival (332). EBV also induces RIPK3 promoter hypermethylation to suppress expression (339). Pseudorabies virus VP22 proteins interact with ZBP1, inhibiting RIPK3 recruitment (341). Poxviruses encode E3L proteins that sequester Z-form RNA, preventing ZBP1 activation, and deploy decoy MLKL proteins lacking the cell-membrane binding domains that sequester RIPK3, preventing cellular MLKL phosphorylation, oligomerization, and permeabilization (329–331). In contrast, norovirus NS3 proteins have repurposed the MLKL executioner domain to actively induce cell death, facilitating viral spread (429). Similarly, IAV NS1 invokes necroptosis by enhancing interactions with RIPK3 phosphorylators, facilitating MLKL oligomerization to regulate viral egress (328, 430, 431). Interestingly, a selective RIPK3 inhibitor, UH15-38, has shown therapeutic promise against IAV *in vivo*, as it blocked IAV-triggered necroptosis, prevented inflammation, and improved animal survival (432). This example illustrates how our understanding of virus-cell death pathway interactions may form the basis for the rational design of novel antiviral therapeutic strategies.

Although IFN-I is unnecessary to initiate programmed cell death in most cell types, IFN-I signaling can synergize with other inflammatory pathways to promote cell death. For example, intracellular PRR sensing of DNA and RNA leads to the activation of RIPK3 in bone marrow-derived macrophages, though this necroptosis response in these macrophages requires cooperative signaling between IFN-I and TNF-α (433). Furthermore, IFN-I signaling can induce expression of cell death-associated factors and sensitize cells to the activation of infection-triggered cell death pathways, illustrating the importance of cross-talk between these antiviral defenses (434, 435).

## CONCLUSIONS AND FUTURE DIRECTIONS

Since its discovery over 60 years ago (436), the IFN-I response has been the focus of antiviral immunity studies in mammals. However, the examples above clearly illustrate that mammals also employ IFN-I-independent antiviral responses that play crucial, and often complementary, roles in controlling viral replication, particularly during early stages of infection or in cell types with limited IFN-signaling capacity. These defenses include cell intrinsic restriction pathways such as autophagy that can degrade incoming virions (119), as well as constitutive factors and systems such as TRIM7 (58, 59, 62, 66) and ERASE (219) that target viral proteins or nucleic acids, respectively, for destruction. Even if viruses can complete their replication, additional IFN-I-independent host defense mechanisms exist to prevent their spread to the next cell, such as the entrapment of exiting viral particles by septins (239, 240) and the induction of cell death pathways (247). It is important to reiterate that many other known IFN-I-independent responses that could not be discussed in detail here, such as various metabolic reprogramming-mediated defenses that create an unfavorable intracellular environment for viral replication, also play critical roles in antiviral immunity (437) (see also Table 1). Thus, together, these IFN-I-independent defenses provide a multilayered response to restrict viral replication, helping to control viral infection when IFN signaling is compromised by viral antagonism or kinetically delayed. However, many key questions regarding the molecular mechanisms governing the activation, regulation, and cell/tissue specificity of these IFN-I defenses and pathways remain. Moreover, a deeper understanding of the cross-talk (if any) between these pathways and the canonical IFN-I response, whether synergistic, redundant, or antagonistic, requires further investigation. Evidence suggests that low-level, constitutive (or “tonic”) IFN-I signaling may help to maintain immune homeostasis by regulating other immune pathways (438–440). Therefore, if, for example, IFN-I signaling maintains basal expression of key components of IFN-I-independent pathways, this could result in important cross-talk between IFN-I-dependent and -independent responses.

New approaches will be needed to specifically identify IFN-I-independent antiviral defenses. High-throughput approaches such as genome-wide CRISPR activation screens have already proven useful in the identification of novel IFN-I-independent antiviral factors, including TRIM7, as well as unappreciated host factors regulating basal and inducible ISG expression. However, such screens on their own do not *per se* identify only IFN-I-independent responses and, in fact, are often swamped by hits related to the IFN-I response. However, combining such screens with IFNAR^-/-^ or STAT1^-/-^ cells may be a powerful strategy to more specifically identify antiviral defense factors that function independently of IFN-I signaling. Moreover, given the fact that viral IFN-I antagonists have revealed key facets of IFN-I response components and regulation (10), viral antagonists might also be useful tools for identifying and studying IFN-I-independent responses. For example, by screening for mammalian poxvirus-encoded factors that retain immunosuppressive function in insect cells (which lack IFNs), poxvirus A51R proteins were discovered as novel immune evasion proteins that rescue the restricted replication of heterologous viruses (80). Follow-up studies aimed at understanding A51R-mediated immune suppression led to the discovery of the FEAR pathway (74), which appears to be conserved between mammals and insects (73). Thus, IFN-I-defective mammalian cells, invertebrate host models, and viral antagonist screens may provide complementary approaches to uncovering new IFN-I-independent defenses.

The recent finding that TRIM7^-/-^ mice are equally susceptible to MNoV infection as wild-type animals underscores the importance of studying IFN-I-independent antiviral factors and pathways identified from cell culture studies in animal models to assess their physiological relevance to controlling viral replication *in vivo*. Studies with TRIM7^-/-^ mice will be needed to determine the *in vivo* role of this host factor in the restriction of other viruses identified as being restricted by TRIM7 in cell culture studies, such as enteroviruses (58, 63). Although several of these recently described IFN-I-independent mechanisms may depend upon host factors that are essential for mouse development (e.g., septins), the use of alternative approaches, such as conditional knockout/knockdown mice (441), may provide strategies for examining their role in antiviral defense in animals.

In conclusion, IFN-I-independent antiviral defenses represent vital, though historically underappreciated, components of the mammalian innate immune response. A deeper understanding of how these defenses are regulated, and how viral pathogens engage with them, will not only lead to a more complete understanding of innate immunity but may also provide new therapeutic strategies to augment their activity for the treatment of viral disease. Development of such approaches could especially benefit individuals with genetic deficiencies in IFN responses, who are predisposed to viral infection (6, 9). Furthermore, understanding IFN-I-independent responses in mammals may also provide critical insights into how lower eukaryotes that lack IFNs combat viral infection. Such knowledge may be useful for the development of intervention strategies to block viral replication in insect vectors that transmit viral pathogens to mammalian hosts. Thus, with the growing appreciation for, and interest in, IFN-I-independent responses, this is an exciting time to explore the additional layers of innate defenses employed by mammals that go beyond IFNs.
